# 1289. Assessing the Safety and Efficacy with Prolonged Use of Tedizolid in Orthopedic Patients

**DOI:** 10.1093/ofid/ofad500.1128

**Published:** 2023-11-27

**Authors:** Daniel E Garza, Christina Maguire, Amanda Binkley, Erica J Weinstein, Randi Silibovsky

**Affiliations:** Penn Presbyterian Medical Center, Philadelphia, Pennsylvania; Penn Presbyterian Medical Center, Philadelphia, Pennsylvania; Penn Presbyterian Medical Center, Philadelphia, Pennsylvania; Perelman School of Medicine at the University of Pennsylvania, Philadelphia, Pennsylvania; Perelman School of Medicine at the University of Pennsylvania, Philadelphia, Pennsylvania

## Abstract

**Background:**

Bone and joint infections (BJI) often require 4 to 6 weeks of treatment with oral or intravenous antimicrobials. To aid in achieving clinical cure, oral agents should be highly bioavailable, have a wide therapeutic index, and ideally penetrate bone tissue well. Data is available for linezolid’s use in BJIs but uptake is limited given its propensity to cause thrombocytopenia with long term use. Tedizolid, however, is associated with less side effects but little remains known about its use for longer treatment durations in BJIs.

**Methods:**

This study is a single center, retrospective cohort study. It evaluated individuals 18 years or older that received tedizolid for 14 days or more for treatment or suppression of BJIs with available follow up lab data monitoring between 2014 and 2022. The primary outcome was occurrence of grade 2 or higher hematological or hepatic National Institute of Health defined adverse drug events during the course of therapy. Secondary outcomes included treatment success defined by no recurrence of infection within 3 months of completing therapy or sustaining suppressive therapy identified by microbiological, clinical or histological evidence.

**Results:**

Thirty-three patients were included in this study and two (6%) patients experienced a safety event, both due to worsened anemia during treatment. Tedizolid also showed efficacy with 26 (90%) of 29 patients completing treatment without recurrence of infection within 3 months. One (25%) of 4 patients failed suppression therapy but are now re-suppressed on tedizolid.

**Conclusion:**

Tedizolid used for 14 days or greater for BJI treatment or suppression was well tolerated and associated with a favorable treatment outcome. Our findings align with other data available highlighting safety of long-term use of tedizolid for non-orthopedic indications.

**Disclosures:**

**Christina Maguire, PharmD**, Viiv: Advisor/Consultant **Amanda Binkley, PharmD, BCIDP, AAHIVP**, Viiv: Advisor/Consultant

